# A self-cascade nanoCRISPR prompts transcellular penetration to potentiate gene editing and tumor killing

**DOI:** 10.1016/j.apsb.2025.09.004

**Published:** 2025-09-08

**Authors:** Chao Liu, Yangsong Xu, Ning Wang, Hongyu Liu, Xi Yang, Shiyao Zhou, Dongxue Huang, Yingjie Li, Yanjie You, Qinjie Wu, Changyang Gong

**Affiliations:** a Department of Biotherapy, Cancer Center and State Key Laboratory of Biotherapy, West China Hospital, Sichuan University, Chengdu 610041, China; b Department of Gastroenterology, People's Hospital of Ningxia Hui Autonomous Region, Yinchuan 750002, China

**Keywords:** CRISPR/Cas9, Gene editing, Nanoplatform, Transcellular penetration, Cancer therapy, Delivery system, Apoptosis

## Abstract

CRISPR/Cas9-based therapeutics face significant challenges in penetrating the dense microenvironment of solid tumors, resulting in insufficient gene editing and compromised treatment efficacy. Current nanostrategies, which mainly focus on the paracellular pathway attempted to improve gene editing performance, whereas their efficiency remains uneven in the heterogenous extracellular matrix. Here, the nanoCRISPR system is prepared with self-cascading mechanisms for gene editing-mediated robust apoptosis and transcellular penetration. NanoCRISPR unlocks its self-cascade capability within the matrix metallopeptidase 2-enriched tumor microenvironment, initiating the transcellular penetration. By facilitating cellular uptake, nanoCRISPR triggers robust apoptosis in edited malignancies, promoting further transcellular penetration and amplifying gene editing in neighboring tumor cells. Benefiting from self-cascade between robust apoptosis and transcellular penetration, nanoCRISPR demonstrates continuous gene transfection/tumor killing performance (transfection/apoptosis efficiency: 1st round: 85%/84.2%; 2nd round: 48%/27%) and homogeneous penetration. In xenograft tumor-bearing mice, nanoCRISPR treatment achieves remarkable anti-tumor efficacy (∼83%) and significant survival benefits with minimal toxicity. This strategy presents a promising paradigm emphasizing transcellular penetration to enhance the effectiveness of CRISPR-based antitumor therapeutics.

## Graphical abstract

**Figure:**
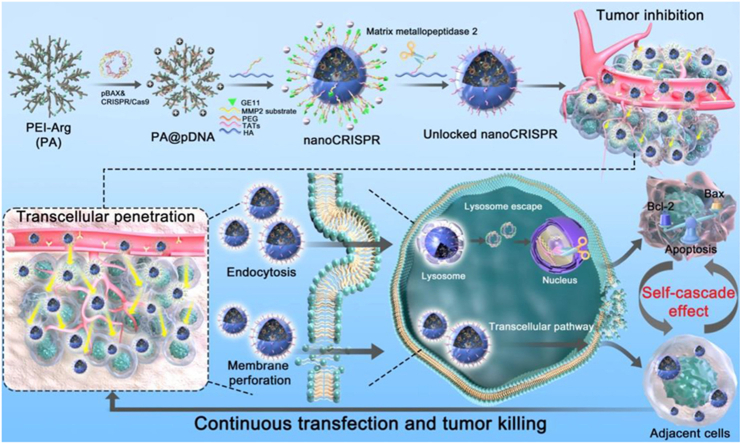

## 1 Introduction

CRISPR/Cas9 technology presents considerable promise in tumor therapy, owing to its precise targeting and editing capabilities within genomes^1^^,^^2^. Through targeted gene editing, the potential disruption of oncogenes or driver genes could facilitate cell death in malignancies. An increasing number of studies have found that CRISPR/Cas9 can be used for *in vivo* tumor treatment by directly editing the genome of cancer cells with the aid of delivery vectors^3^^,^^4^. Especially for undruggable targets, the superiority of CRISPR/Cas9 is emphasized^5^^,^^6^. Despite these efforts, CRISPR-based therapeutics still faces limitations due to the heterogeneous tumor stroma microenvironment *in vivo*, resulting in uneven editing efficiency and therapeutic efficacy remaining far below expectations^7^.

The abnormal extracellular hurdles and the intracellular sequestration constitute the thorny barriers of dense tumor microenvironment^8^. On the one hand, proliferating cancer cells exhibit heterogeneous blood vessel distribution and abnormal blood flow patterns, resulting in uneven perfusion and oxygenation across the tumor mass^9^, ^10^, ^11^. Regions of hypoxia and necrosis further complicate nanomedicine penetration, as these areas may be less accessible to circulating nanoparticles due to poor vascular perfusion and increased interstitial pressure^12^, ^13^, ^14^. Moreover, the tightly compressed tumor cells, acts as a physical barrier, impeding the diffusion and movement of nanomedicine^15^. On the other hand, even if nanomedicines successfully penetrate the tumor interstitium, lysosome is able to capture and degrade CRISPR/Cas9 drugs that enter the cell, compromising the overall gene editing efficiency within tumor^16^. These hurdles lead to uneven distribution of CRISPR/Cas9, which is manifested that gene editing-mediated tumor killing mainly in superficial cells and has a compromise effect on deep tumors^17^. Therefore, it is imperative to improve tumor penetration of CRISPR/Cas9 to elevate gene editing efficacy, thus unleashing the potential of CRISPR/Cas9 in clinical therapeutics.

With the support of nanotechnology, current studies have attempted to overcome the dilemma through intercellular penetration^18^, ^19^, ^20^. Refining the size or shape of nanomedicines facilitates their infiltration into the interstitial spaces of tumor tissues^21^, ^22^, ^23^, ^24^, ^25^. Similarly, surface modifications involving functional groups can modify their charge, hydrophilicity, or hydrophobicity, improving their tissue penetration capabilities^26^, ^27^, ^28^. However, variations in cell density and integrity across different regions of the tumor can lead to uneven distribution of these nanosystems that rely on intercellular pathway for penetration, limiting the permeability of drugs^29^^,^^30^. While directly disrupting or loosening the extracellular matrix may inadvertently disrupt tumor integrity, thereby promoting tumor infiltration and metastasis^31^, ^32^, ^33^, ^34^. Hence, addressing heterogenous matrix microenvironment-related challenges requires innovative design for CRISPR/Cas9-based nanosystem that are superior to tumor penetration.

**Figure 1:**
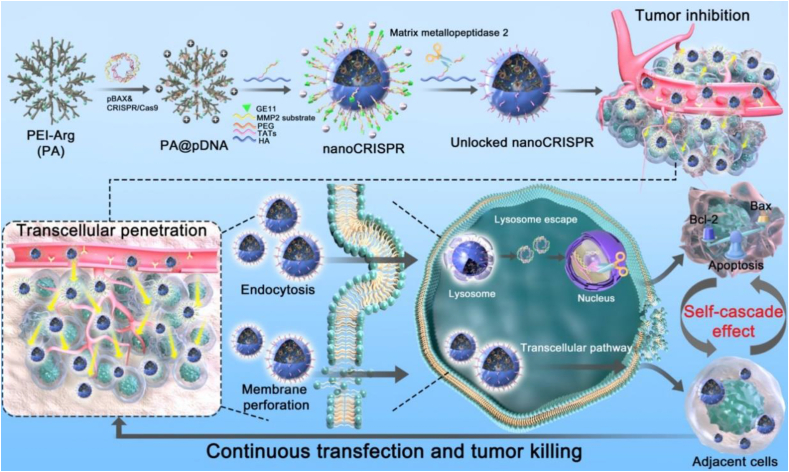
A self-cascade nanoCRISPR is designed for self-synergistic amplification of gene editing-mediated robust apoptosis and transcellular penetration. Benefit from self-cascade capability, nanoCRISPR exhibited continuous gene transfection/tumor killing performance (transfection/apoptosis efficiency-1st: 85%/84.2%, 2nd: 48%/27%) and homogenous penetration *in vitro,* while achieving superior anti-tumor efficacy (∼83%) *in vivo*.

Herein, we constructed nanoCRISPR, a self-cascade nanoCRISPR system designed for enhanced gene editing and tumor eradication *via* transcellular penetration (Fig. 1). Leveraging its extended blood circulation and precise tumor targeting *via* PEGylated shell and GE11-modified HA backbone, nanoCRISPR activates its self-cascade mechanism upon encountering the MMP2 enzyme-rich tumor microenvironment. This mechanism facilitates dual internalization pathways: CD44 receptor-mediated endocytosis induces a proton sponge effect in endo/lysosomes, releasing CRISPR/Cas9-Bcl-2 (pCRISPR) and Bax plasmids (pBax) for apoptosis induction, while cell membrane perforation allows transcellular penetration into neighboring tumor cells. This self-cascading effect between gene editing-mediated apoptosis and transcellular penetration demonstrates potent anti-tumor efficacy, offering a novel perspective to potentiate CRISPR/Cas9-based therapeutics for clinical applications.

## 2 Materials and methods

### 2.1 Chemical reagents

Alfa Aesar (USA) supplied branched PEI 25K and 1.8K. Sodium hyaluronate (HA, *M*_w_: 35 kDa) was provided by Freda Biochem Co., Ltd. (Shandong, China). JenKem Technology Co., Ltd. (Beijing, China) supplied the NH2-PEG_2000_-maleimide. NH_2_-TATs-MMP2-responsive substrate-Cys (YGRKKRRQRRRGPLGIAGQC), NH_2_-TATs-SH (CYGRKKRRQRRR) and Boc-GE11-COOH (YHWYGYTPQNVI) were synthesized by Chinapeptides Co., Ltd. respectively (Shanghai, China). EdU Detection Kit was supplied by RIBOBIO Co., Ltd. (Guangzhou, China). Vazyme Biotech Co., Ltd. (Nanjing, China) provided TUNEL Assay Kit. YOYO-1(C10337, Thermo Fisher, MA, USA), Lipo 3000, TOTO-3(T3604, Thermo Fisher, MA, USA), Hoechst 33342, and LysoTracker Red were purchased by Invitrogen (USA). The antibodies including anti-Bcl-2, anti-Bax, anti-Cleaved Caspase 3 (CC3), and anti-Cleaved Caspase 9 (CC9) were supplied by Abcam (182858, 32503, 32042, 20750S, Abcam, Cambridge, UK). Horseradish peroxidase (HRP)-labeled goat anti-mouse/rabbit antibody and donkey anti-goat secondary antibody were provided by CST (USA).

### 2.2 Cell lines and animals

Human cervical carcinoma (HeLa) and human embryonic kidney cell lines (HEK-293T) were provided by American Type Culture Collection (ATCC; USA). Cell culture media, fetal bovine serum (FBS), penicillin-streptomycin were purchased from Gibco (USA).

HFK Bioscience Co., Ltd. (Beijing, China) supplied the female Balb/c nude mice (5–6 weeks). Pathogen-free mice were utilized for the study of the biodistribution and therapeutic effect. All experimental procedures were executed according to the protocols approved by Sichuan University (Chengdu, PRC) institutional animal care and treatment committee.

### 2.3 Synthesis of PA

Arginine-modified PEI (PA, the core of nanoCRISPR) were achieved by amidation of the amino acid of arginine with the amino group of polyethyleneimine (PEI 1.8K). Briefly, the carboxyl groups of arginine (30 mg) were activated by EDCI and NHS in DMF. We then stirred PEI 1.8K (200 mg) in DMSO for 48 h with Arg-CO-NHS as a subsequent step. Analyses of ^1^H NMR spectroscopy and FT-IR were carried out on the products following dialysis and lyophilization.

### 2.4 Synthesis of HTMPG

Firstly, NH_2_-TATs-MMP2 substrate-Cys (26.6 mg) and MPEG_2000_-maleimide (64.5 mg) were dissolved in PBS and stirred for 24 h. Then, the products were dialyzed against PBS for 72 h. Secondly, the carboxyl groups of HA (85.6 mg) were activated by EDCI and NHS in MES buffer and followed by adding TATs-MMP2 substrate-MPEG_2000_ to obtain HA-TMP. Then, the mixture was dialyzed against PBS for 72 h. Additionally, GE11-COOH (15.8 mg) were also activated using the abovementioned method. After 48 h of reaction (NH_2_-GE11 and HA-TMP), the products were dialyzed and then lyophilized. The obtained HTMPG (the shell of nanoCRISPR) polymer was characterized by ^1^H NMR spectroscopy and FT-IR. HTPG and HTMP which lacked enzymatic sensitivity or tumor target peptide (GE11) were synthesized respectively.

### 2.5 Preparation and characterization of nanoCRISPR

NanoCRISPR consisted of two parts: a cationic core PA and functional corna HTMPG. Firstly, PA/pDNA (mass ratio, 10:1) were gently mixed and co-incubated for 20 min to attain polyplex. Subsequently, HTPG, HTMP or HTMPG was incubated with the above compound for another 30 min to form nanoCRISPR^locked^, nanoCRISPR^-GE11^ or nanoCRISPR (HTMPG: PA: pDNA = 20:10:1). Finally, the ZEN 3690 zetasizer (Malvern, UK) was utilized to measure the particle size and zeta potentials of the nanocomplex. Meanwhile, TEM (JEOL JEM-100CX, Japan) was used to observe and record the morphological characteristics of nanoparticles.

MMP2 (5 μg/mL) or HAase (10 μg/mL) was used to verify the conditioned responsiveness of nanoCRISPR. After degradation for 2 h, we characterized the nanoparticles using the same procedure as mentioned above.

The ability of PA to condense the plasmid (pCRISPR, 2 μg) was evaluated by the gel retardation examination. PA@pCRISPR polyplexes were first prepared at mass ratios of 0.5:1, 1:1, 2:1, 5:1, 10:1 and 15:1, respectively. Subsequently, the nanoparticles were electrophoresed in 1% agarose gel for 20 min at 150 V. The plasmid retardation was observed by a UV visualization system (Bio-Rad).

### 2.6 Construction of plasmids

The sgRNA targeting Human *BCL-2* gene sgRNA was designed by the system provided by Feng Zhang's lab (https://zlab.bio/guide-design-resources) and cloned into Linearized PX330 vector using the protocol previously described. The oligonucleotides were provided by from TSINGKE biological technology.

The Human BAX (NM_138761) ORF was cloned into the pCMV6-Entry backbone linearized by the SgfI and MluI endonucleases. The constructed pCMV6-BAX harbouring the Human BAX expression cassette and kanamycin resistance element for positive clone selection.

### 2.7 Cytotoxicity analysis *in vitro*

The cytotoxicity of HTMPG, PA and PEI 25K was determined by MTT assay. Tumor cells were co-incubated with the formulations at different concentrations (range from 0 to 200 μg/mL). HeLa cells were co-incubated for 2 h with MTT (5 mg/mL) after 48 h incubation. After removing the culture medium from the cells, DMSO was added. Finally, an absorbance at 570 nm was measured with a microplate reader (Bio-Rad 680, USA) in order to assess the viability of the cells.

### 2.8 Cellular internalization and intracellular delivery behavior of nanoCRISPR

Firstly, an APC-CD44 antibody was added to HeLa cells for 2 h (Biolegend, USA). The expression of CD44 receptor on HeLa was confirmed by flow cytometry. YOYO-1 fluorescent dye (Green, Thermo Fisher, USA) (1:5000 for 1 h) was used to label the plasmid. After incubating with PEI 25K, nanoCRISPR or nanoCRISPR + MMP2 for 2 h, the HeLa cells were collected for detection of green fluorescent signals in different groups by flow cytometry.

To study the dual internalization patterns, YOYO-1 labeled-nanoCRISPR pretreated with MMP2 was incubated with CD44 inhibitor-treated HeLa cells or CD44 knockout (CD44^ko^) HeLa cells. The fluorescent signal was analyzed by flow cytometry. Furthermore, YOYO-1 labeled-nanoCRISPR pretreated with MMP2 was incubated with wild type HeLa cells at 4 °C. The cellular uptake assays were performed to verify endocytosis-independent cellular internalization.

Next, we used LSM880 confocal fluorescence microscopy (ZEISS, Germany) for intracellular trafficking of nanoCRISPR. We incubated HeLa cells in confocal culture dishes for 24 h. YOYO-1 labelling plasmids (2 μg/mL) were loaded in nanoCRISPR. After incubations of 0.5, 1, 2 and 5 h, the cells were washed in PBS, followed by LysoTracker Red (Beyotime, China) staining. Then, 4% PFA fixation and PBS washing were performed on the stained cells. Then, Hochest (Beyotime, China) dye was used to label the nucleus for 15 min. Ultimately, cells were observed under CLSM.

### 2.9 Transfection efficacy of nanoCRISPR

We cultured HeLa cells for 12 h with DMEM medium in 6-well plates. Treatment with Lipo 3000, nanoCRISPR or nanoCRISPR + MMP2 in DMEM containing gradient concentrations of serum (10%, 20% or 30%) were performed on the cells. The commercial transfection agent Lipofectamine 3000 (Lipo 3000) was chosen as a control. After 24 h incubation, the GFP expression of HeLa cell was imaged by fluorescence microscope and quantified by flow cytometry.

### 2.10 T7E1 and TIDE assay

After transfection of HeLa cells for 48 h, genomic DNA was extracted using TianGen Kit according to the manufacturer's protocol. The PCR was set up using the following primers F: GAACTCAAAGAAGGCCACAATC; R: CCAAGAATGCAAAG CACATCC. The PCR products were then mixed with T7E1 buffer (VIEWSOLID BIOTECH) to form a heteroduplex. Subsequently, the heteroduplex were incubated with T7E1 enzyme. After 30 min incubation, the products after enzyme digestion were subjected to agarose gel electrophoresis. The genome editing efficiency was calculated by the gray levels of DNA strip which were quantified with ImageJ software.

For TIDE analysis, the PCR products were sequenced and then analyzed by TIDE (Tracking of Indels by DEcomposition) program on http://tide.nki.nl as reported previously.

### 2.11 Apoptosis and proliferation assay

Firstly, we cultured HeLa cells for 12 h with DMEM medium in 6-well plates. Subsequently, different treatments including nanoCRISPR@Null, nanoCRISPR@C (only loading pCRISPR/Cas9), nanoCRISPR@B (only loading pBAX), nanoCRISPR or nanoCRISPR + MMP2 were subjected into cells for 72 h. HeLa cells without treatment were used as the negative control (Blank). Specifically, Annexin V-FITC/PI staining Kit (KeyGen, China) was conducted for apoptosis analysis by flow cytometry.

After transfected with different formulations for 72 h, the cells were stained by EdU (5-Ethynyl-2′-deoxyuridine) detection kits according to the operation manual for proliferation assay. The proliferative cells were stained as red color and the nucleus was stained as blue. Photographing and analyzing stained cells by fluorescence microscopy.

### 2.12 Western blot analysis

HeLa cells were treated with indicated formulations for 72 h, followed by lysed with RIPA buffer (Sigma–Aldrich, USA). Thereafter, cellular proteins were extracted. Subsequently, 20 μg protein of different samples was conducted with SDS-PAGE and then transferred to PVDF membranes (Millipore). After incubated with primary antibodies (Cell Signaling Technology, USA), including anti-Cleaved Caspase 9, anti-Cleaved Caspase 3, anti-Bcl-2, anti-Bax, and anti-GADPH at 4 °C for 12 h, we washed the membranes and co-incubated with the secondary antibodies for 40 min at 37 °C. Finally, enhanced chemiluminescence reagents (Millipore, USA) was used as substrate to produced chemical signals.

### 2.13 Biodistribution of nanoCRISPR

The tumor xenograft model was established by subcutaneous inoculation of HeLa cells at a density of 1.5 × 10^7^ cells per mouse (female Balb/c nude mice, 6 weeks old). When the tumor volume reached ∼300 mm^3^, we randomly divided the tumor-bearing mice into three groups (*n* = 3). The plasmids were labelled by TOTO-3. Each mouse was intravenously injected with nanoCRISPR^locked^, nanoCRISPR^-GE11^, or nanoCRISPR. After 12, 24 and 36 h, the mice were anaesthetized to detect the fluorescence signals using IVIS Lumina Imaging system (PerkinElmer, USA). Meanwhile, the fluorescence signals were quantitatively analyzed by IVIS Lumina Living Imaging software (version 4.2).

### 2.14 Off-target analysis

We extracted the genomic DNA from the major organs of mice with different treatment for off-target analysis using TIANamp Genomic DNA kit (TANGEN Biotech, China). Three potential off-target loci (*mBCL-2*/OFF-1/OFF-2) were identified for the following sequencing. The genome DNA (100 ng) was utilized for amplicon specific PCR using specific primers which flanked the three potential off-target locus. Afterwards, all the PCR with equivalent qualities were prepared for the illumina Hiseq PE 250 deep sequencing. The sequencing result was aligned to the reference genome.

### 2.15 Immunohistochemical analysis

The Institute Research Ethics Committee approved the immunostaining analysis of cervical carcinoma tissues from humans. The slides were dewaxed, and then stained by rabbit anti-Bcl-2 and rabbit anti-Bax primary antibody overnight at 4 °C. Subsequently, after washing with PBS for twice, the secondary donkey anti-rabbit antibody was incubated with the slides for another 1 h at 37 °C. After chemoluminescenced with DAB kit, the images were observed by the microscope (×400).

### 2.16 Antitumor evaluation *in vivo*

In accordance with the above description, tumor xenograft models were established. We randomly divided the mice into seven groups (*n* = 6) when the tumor volume grew to ∼100 mm^3^ and then intravenous administrated with 200 μL of saline or different agents of nanoCRISPR@Null (loading the plasmids encoding nonsense sequences), nanoCRISPR@C (only loading pCRISPR), nanoCRISPR@B (only loading pBAX), nanoCRISPR^locked^, nanoCRISPR^-GE11^ or nanoCRISPR every 3 days. During this period, the length (mm) and width (mm) of the tumors, as well as the body weight of tumor-bearing mice were recorded. In order to calculate the tumor volume, we used the formula, as shown in Eq. (1):

(1) Tumor volume (mm^3^) = *L* × *W*^2^ × 0.5

After treated for 21 days, the mice were killed by cervical dislocation, and the tumors were weighted, photographed. In addition, the organs and tumors were harvested for hematoxylin and eosin (H&E), immunohistochemistry staining (IHC) and TUNEL analysis.

### 2.17 Statistical analysis

GraphPad Prism software (version 8.4.3) was used for statistical analysis. The two-tailed, unpaired Student's *t*-test was used for comparation between two groups. One-way or two-way analysis of ANOVA and Tukey posthoc tests were used when more than two groups were compared. For multiple comparisons, one-way ANOVA followed by Tukey's *post hoc* test was used. For comparation among groups (three or more), one-way ANOVA was utilized. All the data were presented as the mean ± standard deviation (SD) and *P* < 0.05 was considered statistically significant.

## 3 Results and discussion

### 3.1 Synthesis and characterization of nanoCRISPR

**Figure 2:**
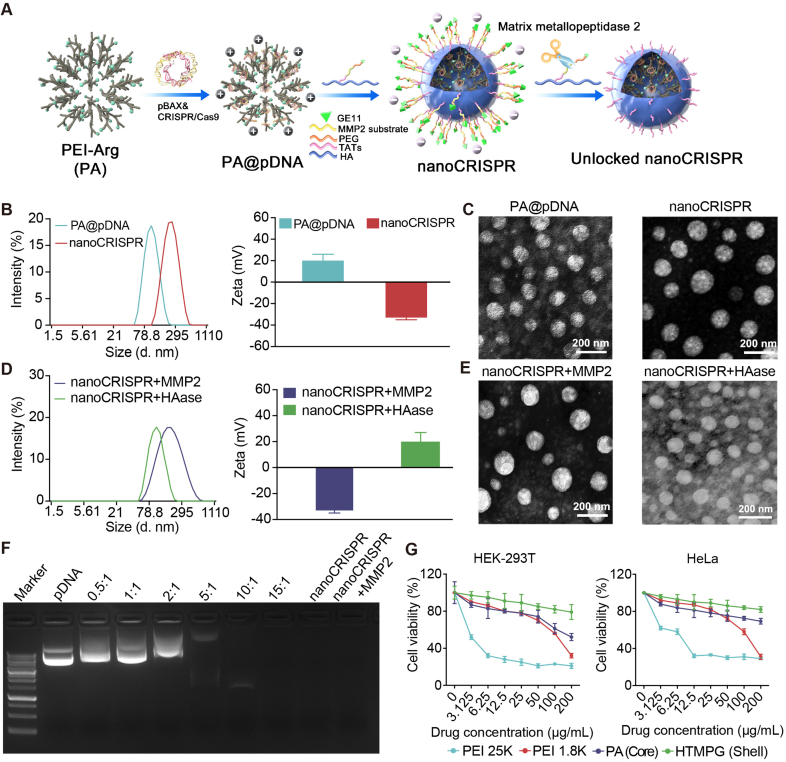
Preparation and characterization of nanoCRISPR. (A) Structure diagram of nanoCRISPR. (B) Dynamic light scattering measurement of nanoCRISPR and PA@pDNA. (C) TEM image of different nanoparticles. (D) Dynamic light scattering measurement of nanoCRISPR in MMP2 or HAase condition. (E) TEM image of nanoCRISPR under MMP2 or HAase condition. (F) Analyzing the ability of nanoparticles to condense pDNA using an agarose gel. The ratio refers to the mass ratio of PA to pDNA (G) MTT assay of different complex against HEK-293T cells and HeLa cells. Data were shown as mean ± SD (*n* = 3 in B–F; *n* = 6 in G).

We firstly synthesized Arginine-grafted polyethyleneimines (PEI) core (PA) for load plasmids. PA was synthesized by polymerization of PEI 1.8K with the arginine, which was verified by ^1^H nuclear magnetic resonance (^1^H NMR) (Supporting Information Fig. S1), indicating the successful synthesis of PA. The ratio of arginine modification was calculated by Ninhydrin analysis. Second, the multifunctional copolymer shell (GE11-PEG-MMP2-TAT-HA, HTMPG) was synthesized (Supporting Information Fig. S2). The characterization of ^1^H NMR and FTIR demonstrated successful synthesis of the shell (Supporting Information Figs. S3 and S4). Subsequently, nanoCRISPR was obtained by coating HTMPG on PA through electrostatic interaction (Fig. 2A). In addition, three control groups were prepared: nanoCRISPR^-GE11^ which lacks tumor targeting peptide, nanoCRISPR^locked^ which lacks MMP2 responsiveness. The dynamic light scattering (DLS) measurement and transmission electron microscopic (TEM) detection of PA@pDNA and nanoCRISPR revealed that the uniformly spherical morphology of PA@pDNA with size of 90 ± 4 nm, and zeta potential on the surface was +28 ± 3 mV (Fig. 2B and C). The hydrodynamic diameter of nanoCRISPR increases to 135 ± 3 nm, and zeta potential converts to −32 ± 3 mV when HTMPG is coated on PA@pDNA. Moreover, we detected the MMP2 and hyaluronidase responsive characteristics of nanoCRISPR by measurement of diameter and surface charge with MMP2 and hyaluronidase incubation respectively. The particle size changed from 135 ± 3 to 110 ± 2 nm, as well the surface charge switched from −32 ± 3 to −36 ± 2 mV in MMP2 condition. In addition, the diameter switched from 135 ± 3 to 95 ± 2 nm, and the zeta potential changed from −32 ± 3 to +27 ± 2 mV, as a result of hyaluronidase mediated degration of the HA backbone of nanoCRISPR (Fig. 2D and E). This result provided evidence of the responsiveness to MMP2 and hyaluronidase.

The agarose gel retardation investigation was conducted to evaluate the plasmid condensation ability of nanoCRISPR (Fig. 2F). With the increasing mass ratios (PA: pDNA), the cumulative plasmid retardation faculty of PA@pDNA was observed. Notably, as with the PA@pDNA at mass ratios above 5:1, nanoCRISPR completely retarded the plasmid, demonstrating excellent encapsulation capacity and protective performance.

Next, we evaluated the cytotoxicity of nanoCRISPR in HEK-293T and HeLa cells by MTT analysis (Fig. 2G). As shown, PA only had a slight effect on the viability of both cell lines. Even at concentrations up to 200 μg/mL, HTMPG copolymer, the corona of nanoCRISPR, did not show obvious cytotoxicity. These results demonstrated that the components of nanoCRISPR have a safety profile for *in vivo* application.

### 3.2 Internalization capacity and transfection efficiency of nanoCRISPR

**Figure 3:**
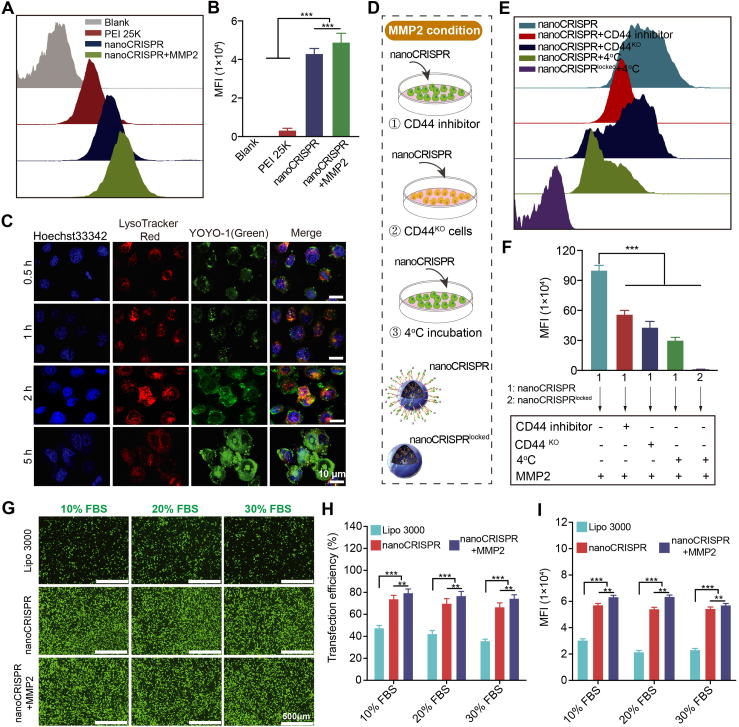
Cellular internalization behavior of HeLa. (A, B) Cellular uptake efficiency by flow cytometry analysis. (C) Subcellular localization at different time points of nanoCRISPR under MMP2 condition by scanning laser microscope. Scale bar: 10 μm. (D) Schematic diagram of experimental procedure to confirm the dual internalization capacity of nanoCRISPR. (E, F) Cellular uptake efficiency under indicated treatments. (G–I) Fluorescence images and flow cytometric analysis of HeLa cells after transfection with Lipo 3000, nanoCRISPR or nanoCRISPR + MMP2 in DMEM containing 10%–30% concentrations of serum. Data were shown as mean ± SD (*n* = 6) and statistically analysed using one-way ANOVA and Tukey's tests. ∗*P* < 0.05, ∗∗*P* < 0.01, ∗∗∗*P* < 0.001.

To estimate the cellular uptake efficacy, we co-incubated YOYO-1 labeled nanoCRISPR (green) with HeLa cells for flow cytometric assay. As shown in Fig. 3A and B, nanoCRISPR group showed a high mean fluorescence intensity (MFI). Notably, nanoCRISPR exhibited much higher MFI in the MMP2 condition. This result demonstrated that the internalization capacity of nanoCRISPR was unlocked and enhanced in simulated TME.

Furthermore, the intracellular delivery behavior of nanoCRISPR was investigated using confocal scanning laser microscopy (CSLM) (Fig. 3C and Supporting Information Fig. S5). By labeling with YOYO-1 (plasmid: green) and LysoTracker (lysosome: red), intracellular tracking revealed that nanoCRISPR gradually penetrated the cell membrane and entered the lysosome within 0.5 to 1 h. Additionally, a portion of nanoCRISPR entered the cytoplasm directly through perforation, bypassing endo/lysosomal traps, which promoted transcellular penetration of nanocomplex. After 5 h of incubation, the green signal was observed in the cytoplasm, and the signal within the lysosome was released *via* the proton sponge effect, entering the nucleus. This suggests that nanoCRISPR enters cells through various manners.

According to our design, nanoCRISPR enters cells through dual internalization modes: (1) CD44 receptor-mediated endocytosis (Supporting Information Fig. S6)^35^. (2) TAT-mediated membrane perforation, which operates independently of endocytosis^36^. To investigate these internalization modalities, we assessed the cellular uptake of HeLa cells treated with nanoCRISPR under various conditions. As depicted in Fig. 3D–F, nanoCRISPR pretreated with MMP2 displayed optimal MFI across all groups. Blocking the CD44 receptor with antagonists resulted in reduced cellular uptake efficiency of nanoCRISPR, consistent with findings in CD44 knockout (CD44^ko^) HeLa cells. To evaluate TAT-mediated internalization, independent of endocytosis, all endocytic pathways of HeLa cells were inhibited simultaneously at 4 °C. Our observations indicated that nanoCRISPR maintained a certain level of cellular uptake efficiency *via* membrane perforation even in the absence of endocytosis. These results suggest that nanoCRISPR undergoes perforation-assisted internalization alongside CD44 receptor-mediated endocytosis. This dual pattern of internalization ensures self-cascade capability and transcellular mechanism within the nanoCRISPR system.

The expression of plasmid and the stability of transfection are crucial steps for realizing the function of CRISPR/Cas9-mediated gene editing. Therefore, we further investigated the transfection of nanoCRISPR under conditions containing different serum concentrations in the medium. Lipofectamine 3000 (Lipo 3000), a widely used commercial transfection reagent, served as the control group for comparison. Quantitative evaluation of EGFP-positive cells by flow cytometry is presented (Fig. 3G–I and Supporting Information Figs. S7–S9). The transfection efficiency of the nanoCRISPR-treated group was significantly higher (∼86%, ∼84%, ∼81%) than that of Lipo 3000 (∼55%, ∼40%, ∼30%) in the medium containing different concentrations of serum, respectively. Additionally, nanoCRISPR pretreated with MMP2 maintained stable transfection performance (∼81%) even at 30% serum concentration, while the transfection efficiency of Lipo 3000 decreased to 30%. By harnessing the dual internalization capacity, nanoCRISPR exhibited optimal transfection efficacy in all groups under simulated tumor microenvironment (TME) conditions (MMP2 condition). These results demonstrate the remarkable serum tolerance and efficacy achieved through nanoCRISPR-mediated transfection.

### 3.3 Self-cascade capability of nanoCRISPR

**Figure 4:**
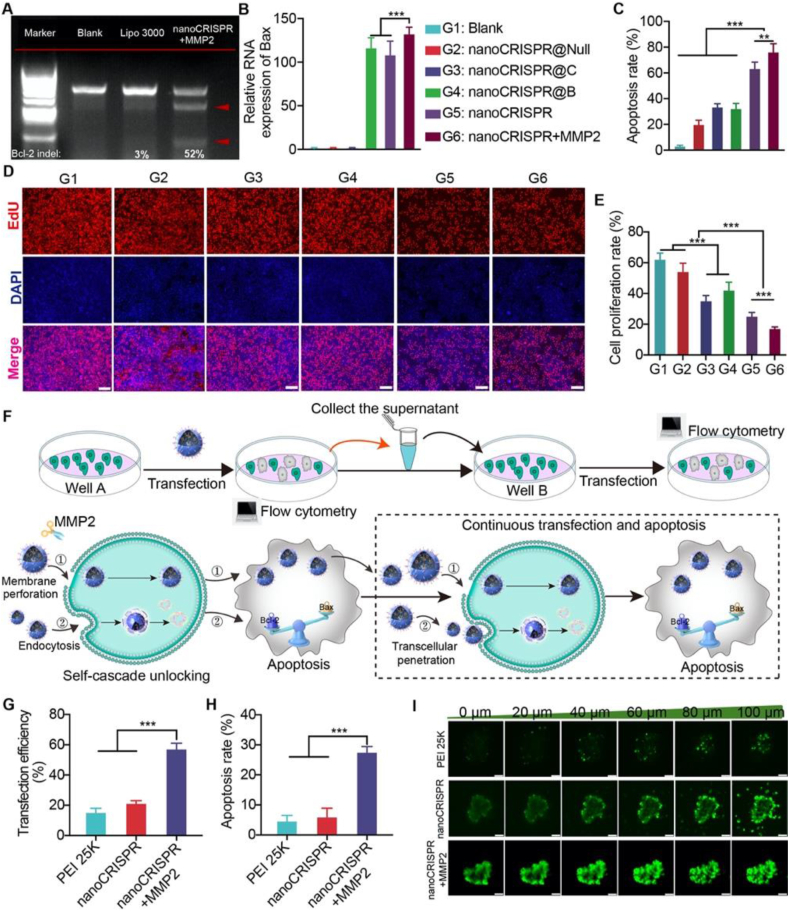
The self-cascade capability of nanoCRISPR. (A) T7E1 assay of Lipo 3000 and nanoCRISPR + MMP2 group, untreated cells were used as control (Blank). (B) Detection of Bax at RNA level. (C) Apoptosis analysis of HeLa cells treated with indicated formulations including Blank, nanoCRISPR@Null (Null: plasmids encoding nonsense sequences), nanoCRISPR@C (only loading pCRISPR/Cas9), nanoCRISPR@B (only loading pBAX), nanoCRISPR and nanoCRISPR + MMP2. (D, E) The cell proliferation of HeLa cells with different treatments. (F) Schematic diagram of study method and mechanism of the self-cascade effect. (G, H) The continuous transfection and apoptosis efficacy of nanoCRISPR. (I) 3D tumor spheroids model to examine the homogeneous penetration performance. The tumor spheroid was scanned from the surface to the middle using a Z-stack scanning technique. Data were shown as mean ± SD (*n* = 6) and statistically analysed using one-way ANOVA and Tukey's tests. ∗*P* < 0.05, ∗∗*P* < 0.01, ∗∗∗*P* < 0.001.

We investigated nanoCRISPR mediated comprehensive regulation of the BCL-2/BAX axis (pCRISPR and pBAX) (Supporting Information Fig. S10). Initially, we assessed the gene editing efficiency of *BCL-2* using T7EI and TIDE analysis^37^. The results revealed that the *BCL-2* editing efficacy in the nanoCRISPR group was approximately 17-fold higher than that in the Lipo 3000 group (Fig. 4A and Supporting Information Fig. S11). Additionally, we evaluated BAX expression at the RNA level using quantitative PCR (Fig. 4B), showing an upregulation of Bax RNA by approximately 130-fold in the nanoCRISPR group compared to the control group. These findings demonstrate the capability of nanoCRISPR to comprehensively regulate the apoptosis pathway through the BCL-2/BAX axis.

Given the effectiveness of BCL-2/BAX axis regulation, we quantitatively investigated apoptosis using annexin V-FITC/PI assay (Fig. 4C and Supporting Information Fig. S12). Due to the comprehensive regulation of the BCL-2/BAX axis, nanoCRISPR treatment led to a significant increase in apoptosis tumor cells compared to groups treated with nanoCRISPR@C (loaded only with pCRISPR/Cas9) or nanoCRISPR@B (loaded only with pBAX), respectively. Moreover, the nanoCRISPR group induced robust tumor apoptosis (∼76%) when exposed to MMP2, highlighting the advantage in tumor simulated condition. Subsequently, we investigated the effect of nanoCRISPR on tumor cell proliferation using EdU assay. NanoCRISPR-treated tumor cells exhibited an approximate ∼80% decrease in proliferation rate under MMP2 conditions, demonstrating the highest efficiency among the groups (Fig. 4D and E). Additionally, we assessed the expression of key proteins involved in apoptosis (BCL-2, BAX, Cleaved caspase 9: CC9, Cleaved caspase 3: CC3) (Supporting Information Figs. S13 and S14). These results suggest that the comprehensive regulation of the apoptosis pathway by nanoCRISPR demonstrates excellent anti-tumor efficacy *in vitro*.

Encouraged by the remarkable anti-tumor efficacy, we delved into the self-cascading effects triggered continuous gene transfection and tumor apoptosis. HeLa cells underwent initial transfection with nanoCRISPR under various conditions for 48 h. Subsequently, we harvested the supernatant for a subsequent round of transfection of untreated cells (Fig. 4F). Transfection and apoptosis efficacy were analyzed by flow cytometry, respectively. When the self-cascading properties of nanoCRISPR remained locked, the efficiency of multi-round transfection and apoptosis was comparable to that of the PEI 25K group (Fig. 4G and H, Supporting Information Figs. S15 and S16). Conversely, nanoCRISPR exhibited the highest sustainable transfection and multi-round apoptosis efficiency (48% and 27%, respectively) under MMP2 conditions among all groups due to its self-cascade effect. The results suggested a self-cascade effect between gene editing mediated apoptosis and transcellular penetration triggered by nanoCRISPR. The superior properties facilitate nanoCRISPR to trigger homogeneous gene editing and antitumor efficacy in solid tumors.

Multicellular spheroids are commonly utilized as 3D *in vitro* models to simulate solid tumor growth *in vivo*. Therefore, we examined the diffusion of nanoCRISPR using 3D tumor spheroid models *via* confocal fluorescence microscopy assay (Fig. 4I). Benefiting from the activation of its self-cascade property, nanoCRISPR pretreated with MMP2 demonstrated the most uniform transfection. Furthermore, quantitative analysis of the fluorescence signal reaffirmed the outstanding penetration capability of nanoCRISPR, implying the advantages of the self-cascade effect in solid tumors.

### 3.4 Biodistribution of nanoCRISPR

**Figure 5:**
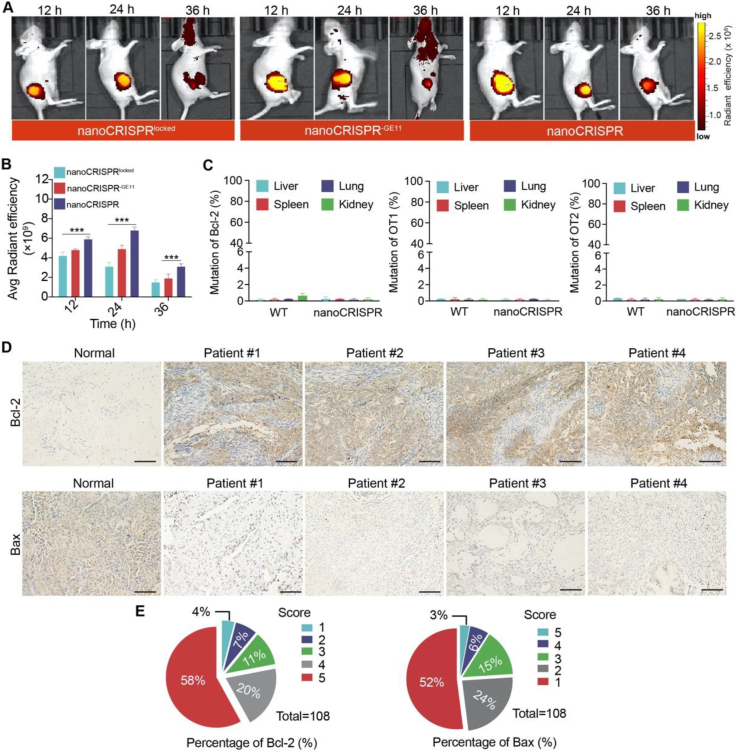
Biodistribution of nanoCRISPR and the analysis of clinical samples. (A) The biodistribution imaging of xenograft tumor-bearing mice at 12, 24 and 36 h after intravenous injection of indicated formulations. (B) The fluorescence intensity statistics of tumors in indicated groups. (C) The mutation of Bcl-2 locus/candidate off-target 1 (OT1)/candidate off-target 1 (OT2) in major organs were analyzed by deep sequencing with illumine Hiseq 2500 PE250. Data were shown as mean ± SD (*n* = 6) and statistically analysed using one-way ANOVA and Tukey's tests. ∗*P* < 0.05, ∗∗*P* < 0.01, ∗∗∗*P* < 0.001. (D, E) Immunohistochemical staining and expression analysis of Bcl-2 or Bax in clinical samples with cervical cancer (scale bars = 100 μm) (*n* = 108).

Achieving precise tumor targeting is a crucial prerequisite for unlocking the self-cascade capability of nanoCRISPR. Hence, we conducted live image analysis to assess the biodistribution of the nanocomplex in a xenograft tumor model. Following intravenous injection of nanoCRISPR, a significant portion of the fluorescent signal was observed to accumulate at the tumor site within 12 h, reaching its peak at 24 h (Fig. 5A and B). In consideration of safety concerns for clinical applications, it is imperative to evaluate potential off-target effects induced by the CRISPR/Cas9 system. Therefore, we analyzed off-target effects in mouse organs through deep sequencing. The results revealed that the mutation frequencies of three potential off-target loci of the *BCL-2* gene in vital organs were all below 1% (Fig. 5C). These negligible mutations occurred at single nucleotide loci, likely attributed to sequencing errors. These findings affirm nanoCRISPR as a safe and precise nanoplatform for *in vivo* tumor treatment.

Moreover, the clinical feasibility of nanoCRISPR-based therapeutics was assessed. A total of 48 human cervical squamous cell carcinoma (CSC) samples was collected for analysis. Immunohistochemistry (IHC) staining was performed to detect the expression of BCL-2/BAX proteins. The findings indicated that in the majority of samples, increased BCL-2 expression coincided with decreased Bax expression, suggesting a mutual compensatory mechanism within the BCL-2/BAX axis contributing to tumor resistance to apoptosis (Fig. 5D and E). This clinical feature highlights the potential limitations of monotherapy targeting a single node of the apoptosis pathway. Additionally, it underscores the promising prospects of nanoCRISPR therapeutics in clinical settings, given its ability to comprehensively regulate the BCL-2/BAX axis.

### 3.5 Anti-tumor effect *in vivo*

**Figure 6:**
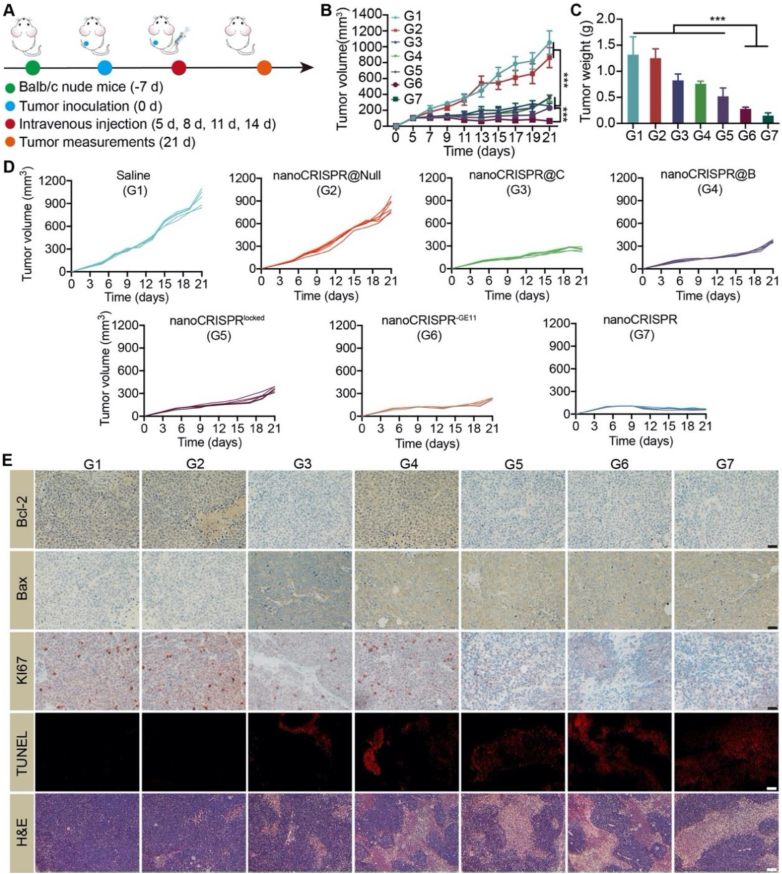
The evaluation of anti-tumor efficacy of nanoCRISPR *in vivo*. (A) Schematic of the treatment in HeLa tumor-bearing mice. (B–D) Summarized/individual tumor growth curves and weight of the mice after treatment with Saline (G1), nanoCRISPR@Null (Null: plasmids encoding nonsense sequences) (G2), nanoCRISPR@C (only loading pCRISPR/Cas9) (G3), nanoCRISPR@B (only loading pBAX) (G4), nanoCRISPR^locked^ (lacks MMP2 responsiveness) (G5), nanoCRISPR^-GE11^ (lacks tumor targeting peptide) (G6), nanoCRISPR (G7). (E) IHC and H&E staining of tumor sections collected after various treatments for 21 days (scale bars = 20 μm). Data were shown as mean ± SD (*n* = 6) and statistically analysed using one-way ANOVA and Tukey's tests. ∗*P* < 0.05, ∗∗*P* < 0.01, ∗∗∗*P* < 0.001.

Building upon the remarkable tumor targeting and homogeneous diffusion capabilities of nanoCRISPR, we conducted further investigations into its therapeutic efficacy using a xenograft tumor model (Fig. 6A). As illustrated in Fig. 6B–D and Supporting Information Fig. S17, tumors treated with saline (1056 ± 164 mm^3^) and nanoCRISPR@Null (863 ± 126 mm^3^) exhibited uncontrolled progression. In contrast, treatment with nanoCRISPR, leveraging its self-cascade capability, demonstrated the most robust inhibition of tumor growth across all groups (65 ± 6 mm^3^), outperforming both the nanoCRISPR@C (258 ± 45 mm^3^) and nanoCRISPR@B (363 ± 36 mm^3^) monotherapy groups. This finding was further supported by the assessment of isolated tumor weight. These results indicated the excellent anti-tumor efficacy of nanoCRISPR, validating the advantages of its self-cascade capabilities and comprehensive regulation of the apoptotic pathway.

Moreover, the expression levels of BCL-2 and BAX proteins, along with markers related to tumor proliferation and apoptosis, were assessed through immunohistochemistry (IHC) and immunofluorescence (IF) staining (Fig. 6E and Supporting Information Figs. S18–S21). Tumors treated with saline exhibited elevated BCL-2 expression coupled with decreased BAX expression, indicative of a compensatory mechanism conferring resistance to apoptosis. Remarkably, the nanoCRISPR group demonstrated the lowest BCL-2 expression and highest BAX protein levels among all treatment groups, validating the comprehensive regulation of the BCL-2/BAX axis facilitated by the self-cascade capabilities. Furthermore, the nanoCRISPR group exhibited optimal apoptosis induction and proliferation suppression compared to other treatments. Additionally, blood chemistry analysis and complete blood count results showed no significant differences across all groups, further suggesting the potential clinical applicability of nanoCRISPR (Supporting Information Figs. S22 and S23).

## 4 Conclusions

In this study, we presented a novel self-cascade nanoplatform (nanoCRISPR) to enhance gene editing and tumor inhibition by facilitating transcellular penetration. NanoCRISPR demonstrates remarkable cellular uptake and transfection efficacy in environments mimicking MMP2-rich TMEs. Following nanoCRISPR treatment, we observed a substantial 52% improvement in *BCL-2* gene editing efficiency and an approximately 130-fold upregulation of Bax mRNA expression in tumor cells compared to control groups, leading to comprehensive regulation of the BCL-2/BAX axis and robust tumor apoptosis. More excitingly, nanoCRISPR exhibits continuous gene editing and apoptosis-inducing capabilities, attributed to its self-cascading effects of apoptosis and transcellular penetration. Biodistribution analysis and 3D tumor spheroid assays confirm nanoCRISPR's specific tumor recognition and homogeneous diffusion, culminating in superior anti-tumor efficacy *in vivo* without discernible adverse effects.

The self-cascade design strategy underpinning nanoCRISPR enables sustained circulation of active gene editing and tumor-killing effects, addressing an urgent need in CRISPR-based therapeutics for solid tumors. Prospectively, this work lays the foundation for an innovative and versatile nanoplatform with immense potential for clinical application in challenging solid tumor contexts.

## Author contributions

Chao Liu: Conceptualization, Writing – original draft, Methodology, Investigation. Yangsong Xu: Writing – original draft, Methodology, Investigation, Conceptualization. Ning Wang: Methodology, Investigation. Hongyu Liu: Methodology, Investigation. Xi Yang: Methodology, Investigation. Shiyao Zhou: Methodology, Investigation. Dongxue Huang: Investigation. Yingjie Li: Investigation. Yanjie You: Methodology. Qinjie Wu: Supervision, Writing – review & editing. Changyang Gong: Writing – review & editing, Supervision, Resources, Funding acquisition.

## Conflicts of interest

The authors have no conflicts of interest to declare.

## Appendix A Supporting information

The following is the Supporting information to this article:
