# Supplementary material for: A self-cascade nanoCRISPR prompts transcellular penetration to potentiate gene editing and tumor killing
Source: Acta Pharm Sin B. 2025 Sep 8;15(11):5933–44. doi: 10.1016/j.apsb.2025.09.004 (PMC12648002; doi:10.1016/j.apsb.2025.09.004)
Supplement: Multimedia component 1 [file mmc1.pdf]

Supporting Information for

ORIGINAL ARTICLE

A self-cascade nanoCRISPR prompts transcellular penetration to potentiate gene editing and tumor killing

Chao Liu[a,†], Yangsong Xu[a,†], Ning Wang[a], Hongyu Liu[a], Xi Yang[a], Shiyao Zhou[a], Dongxue Huang[a], Yingjie Li[a], Yanjie You[b], Qinjie Wu[a], Changyang Gong[a,*]

[a]*Department of Biotherapy, Cancer Center and State Key Laboratory of Biotherapy, West China Hospital, Sichuan University, Chengdu 610041, China*

[b]*Department of Gastroenterology, People's Hospital of Ningxia Hui Autonomous Region, Yinchuan 750002, China*

Received 12 February 2025; received in revised form 20 April 2025; accepted 18 June 2025

*Corresponding authors.

E-mail addresses: chygong14@163.com (Changyang Gong).

[†]These authors made equal contributions to this work.

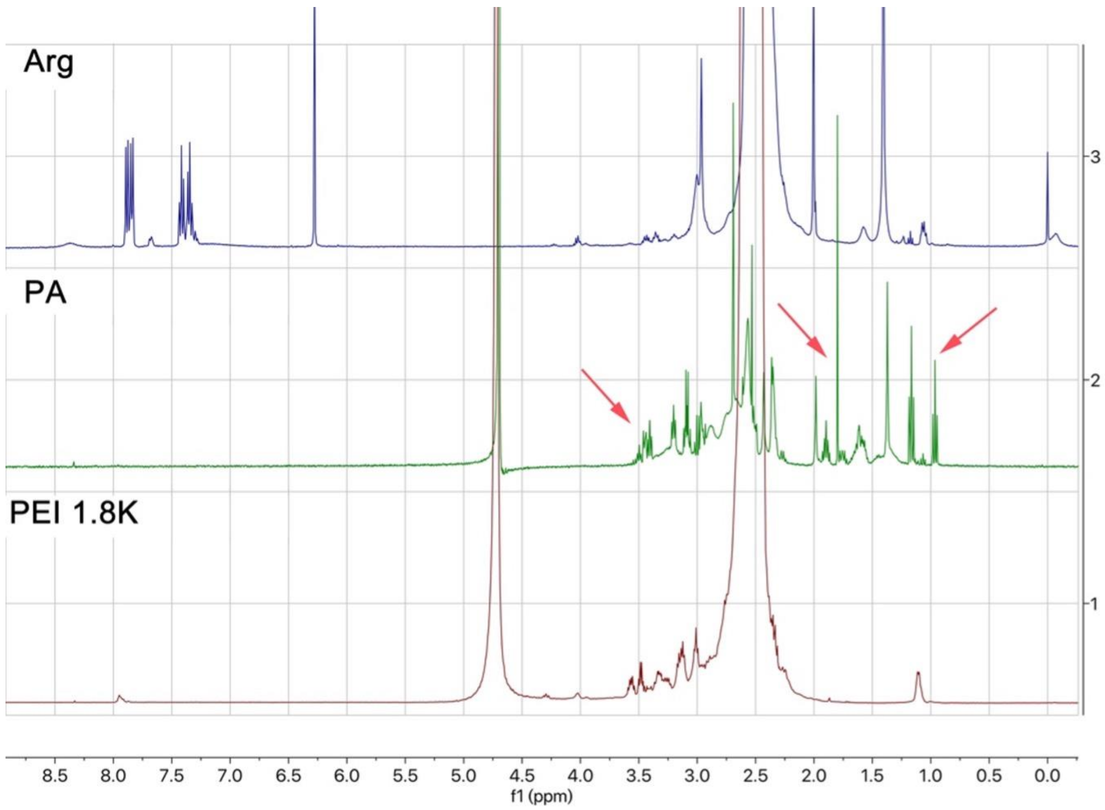

Figure S1 ^{1}H NMR of PA.

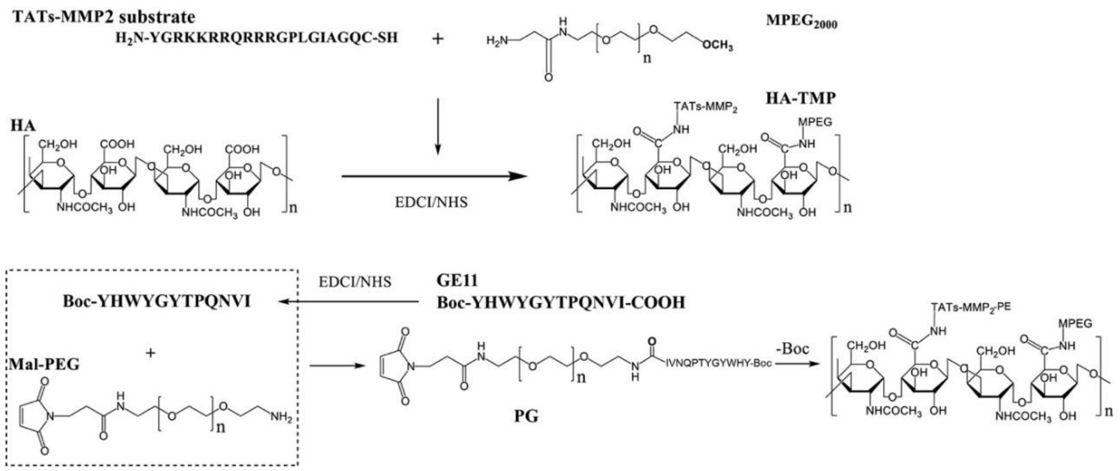

Figure S2 Synthetic routes of HTMPG copolymer.

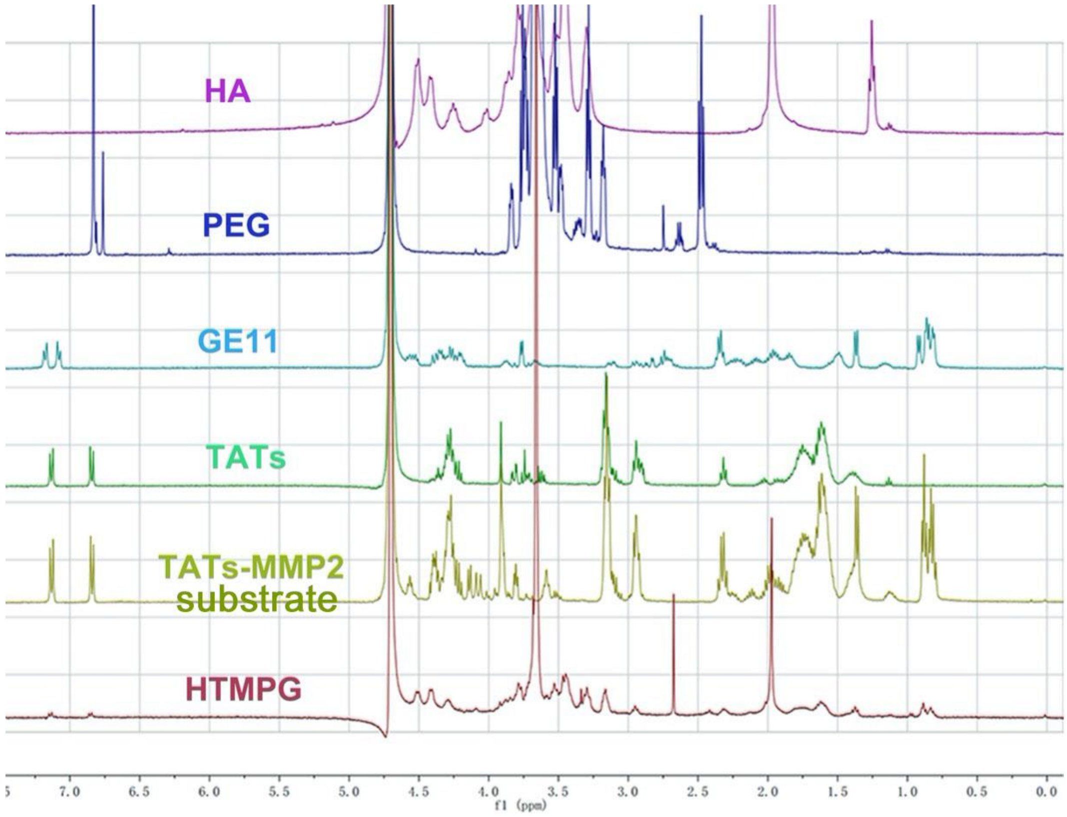

Figure S3 ^{1}H NMR spectrum of HTMPG.

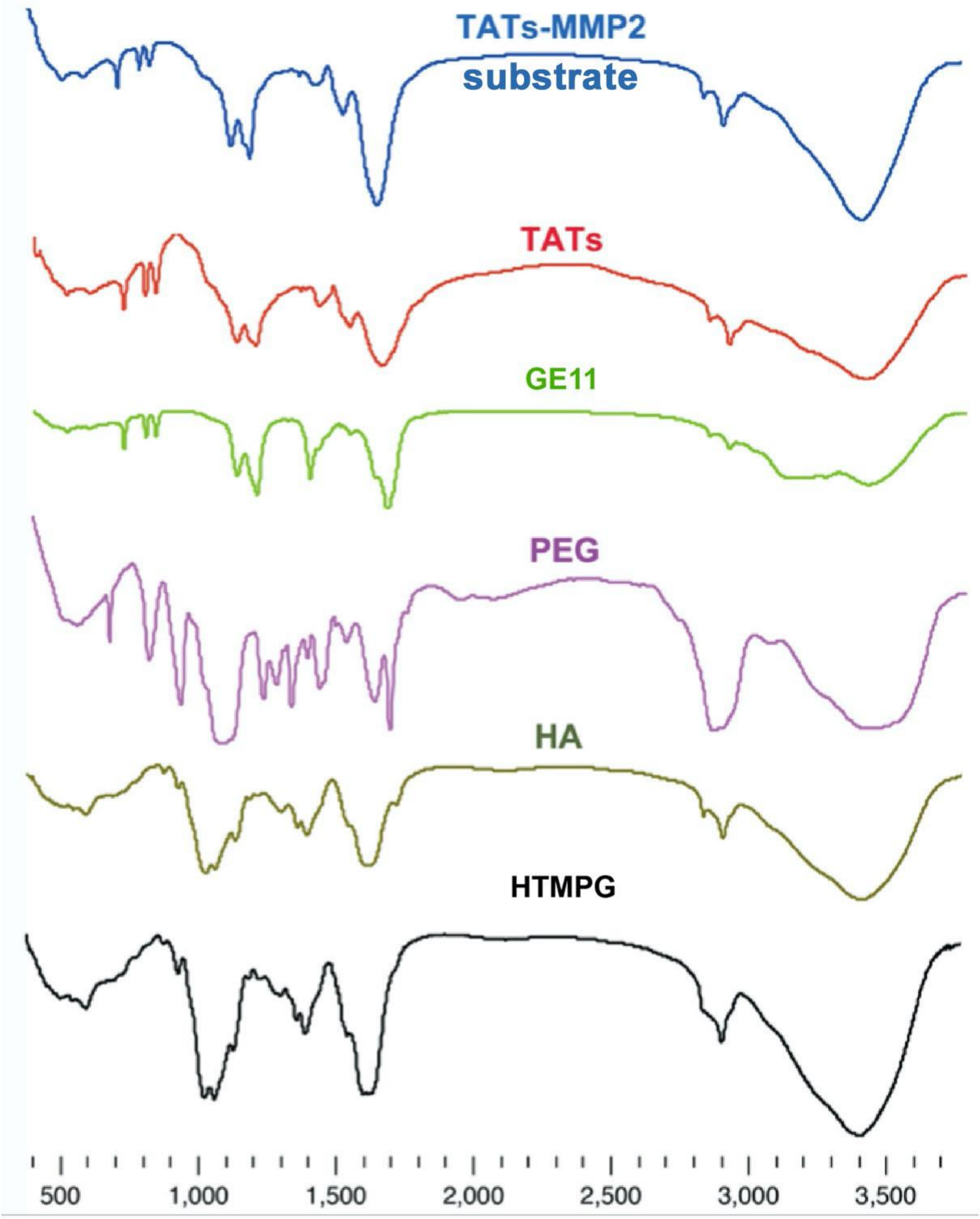

Figure S4 FTIR spectrum of HTMPG.

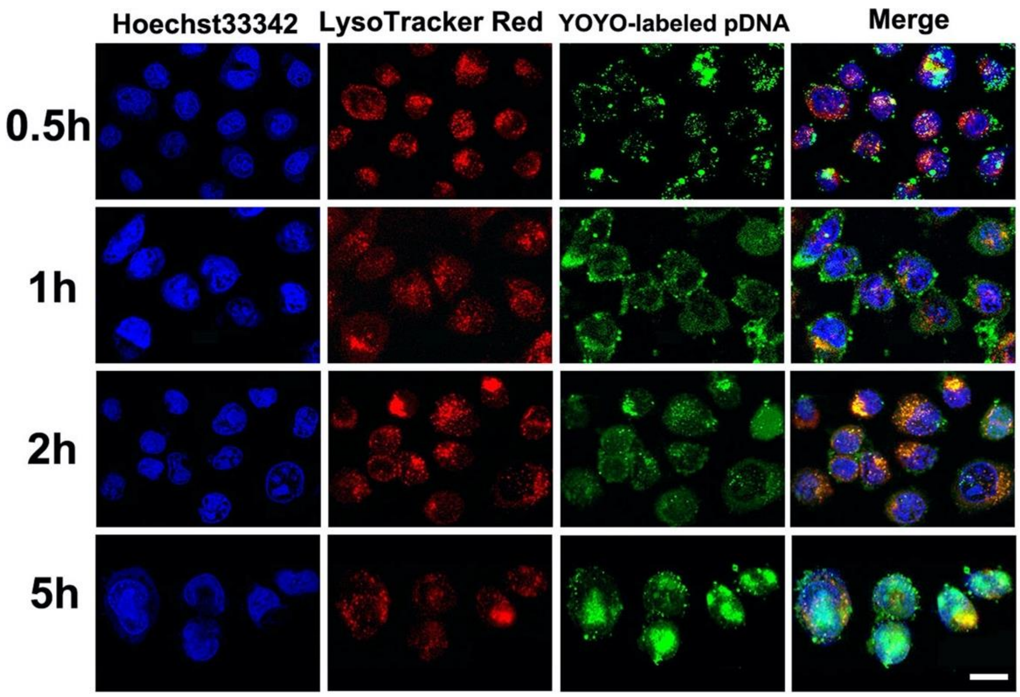

Figure S5 Subcellular localization at indicated time points of PEI 25K.

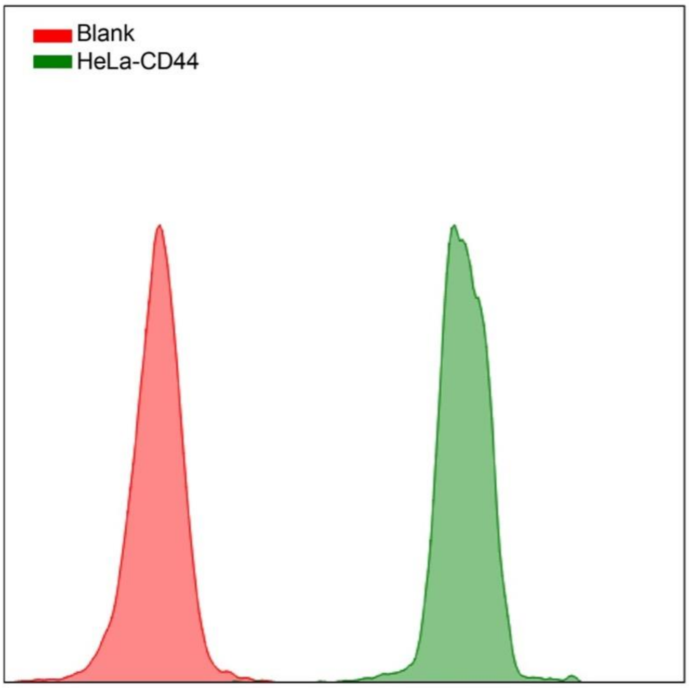

Figure S6 CD44 expression on HeLa cells.

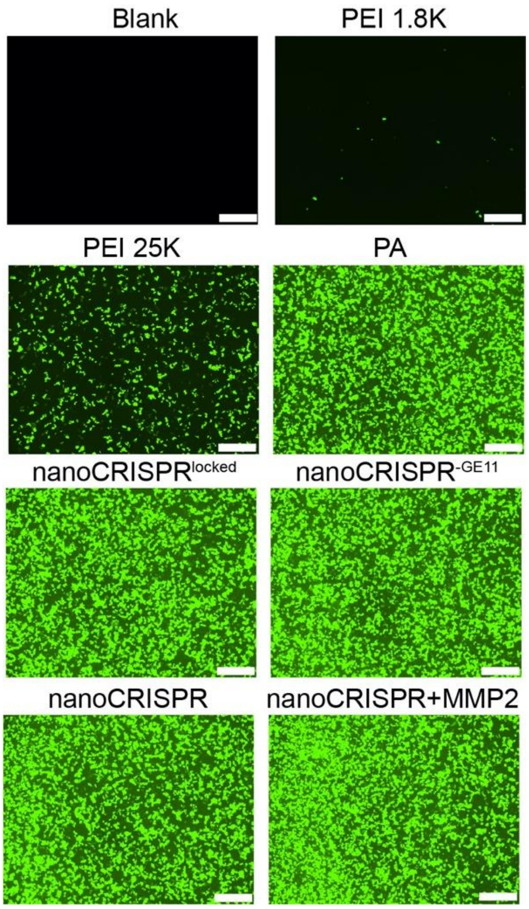

Figure S7 Fluorescence images a of HeLa cells after transfection with indicated formulations.

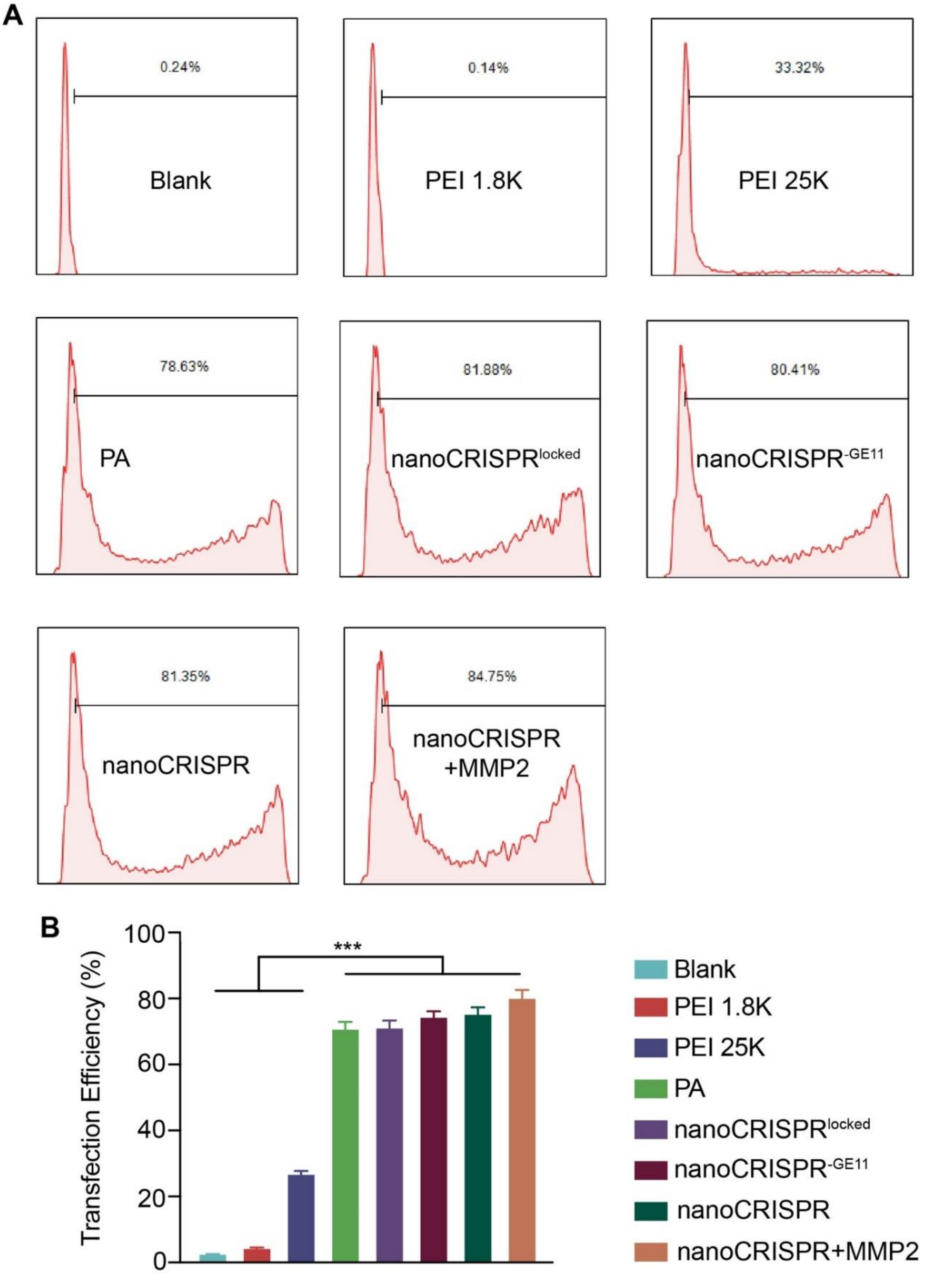

Figure S8 The analysis of transfection efficiency by flow cytometry.

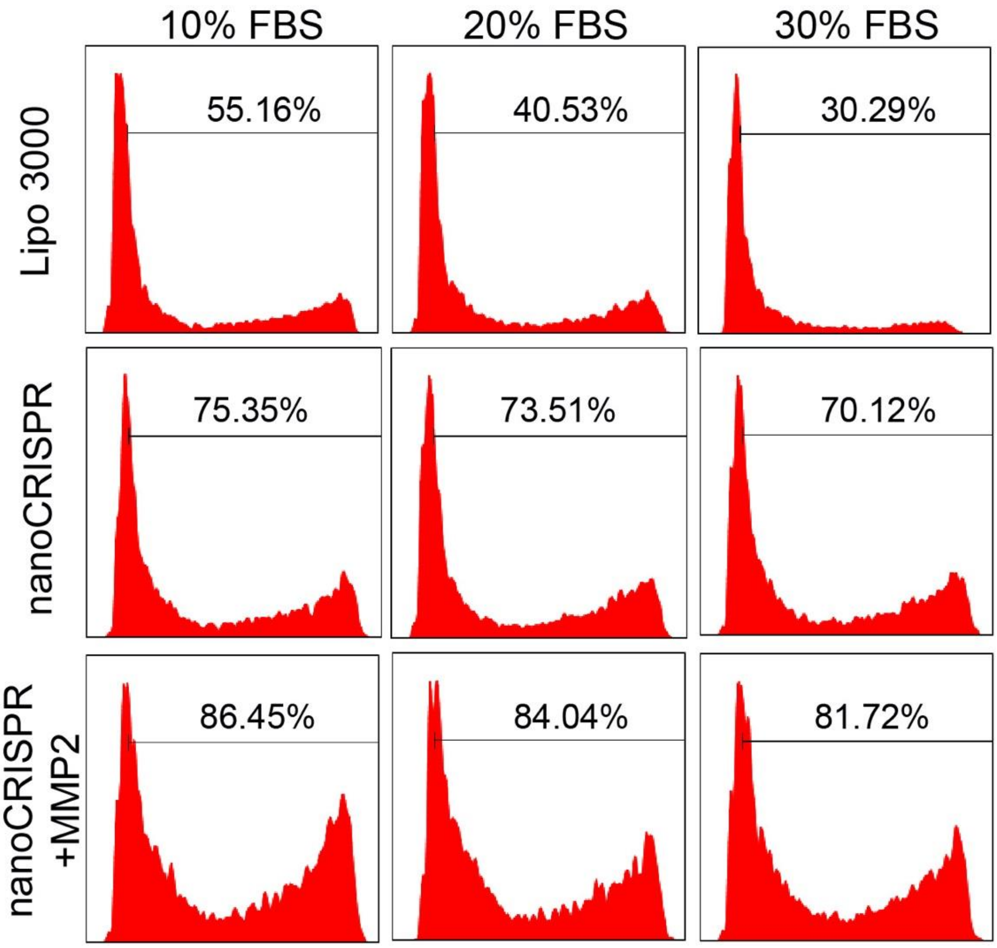

Figure S9 Flow cytometric analysis of HeLa cells after transfection with Lipo 3000, nanoCRISPR or nanoCRISPR+MMP2 in medium containing10%–30% concentration of serum.

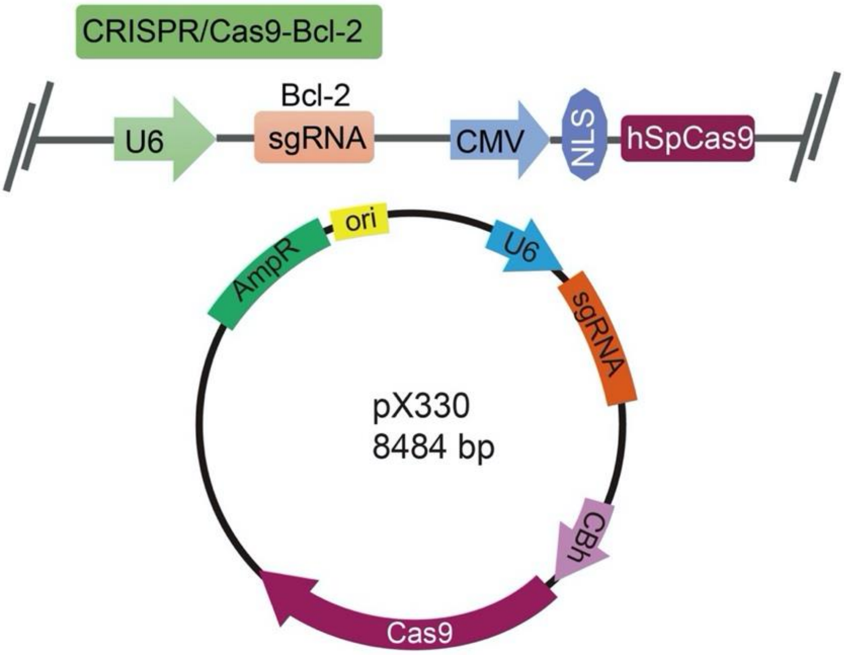

Figure S10 Schematic diagram of CRISRP/Cas9-Bcl-2 plasmid.

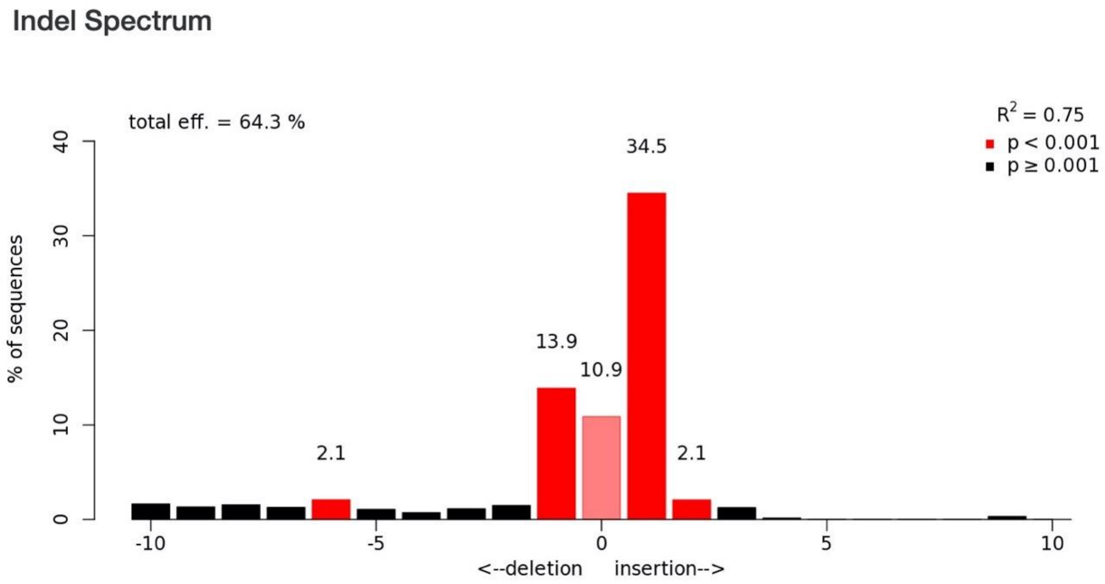

Figure S11 TIDE analysis of HeLa cells with nanoCRISPR+MMP2 transfection.

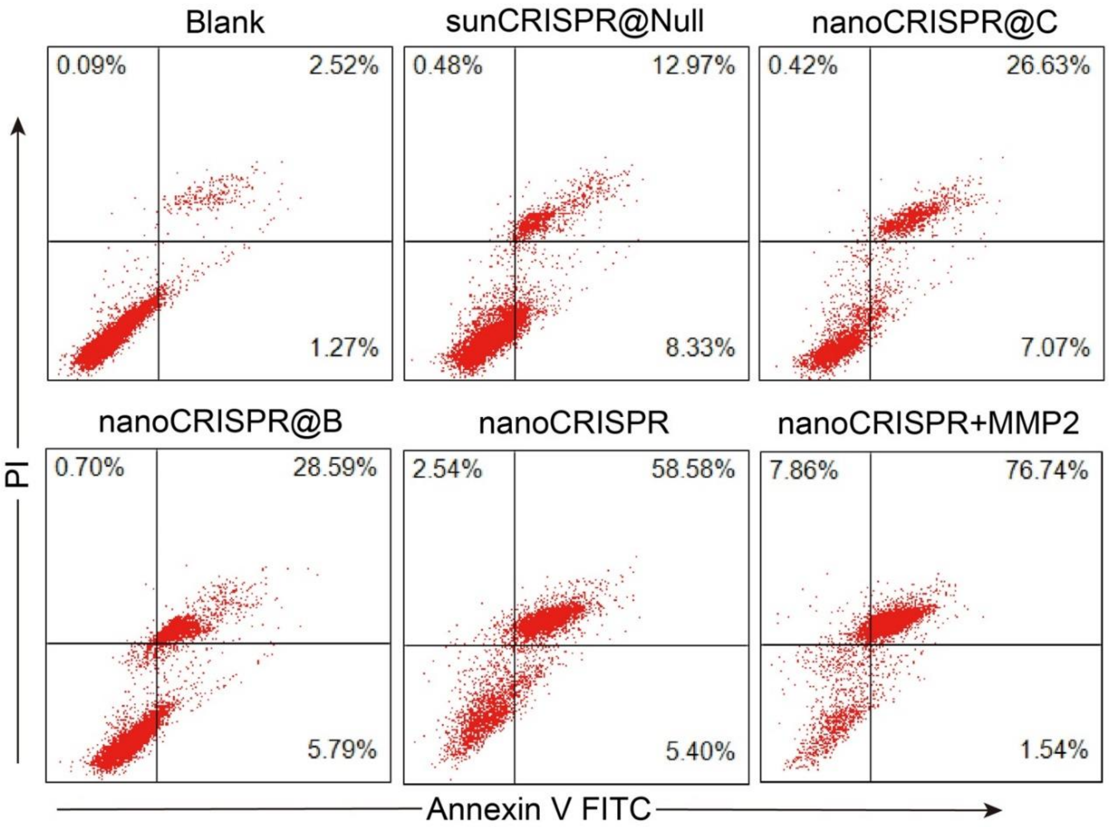

Figure S12 Apoptosis analysis by flow cytometry analysis.

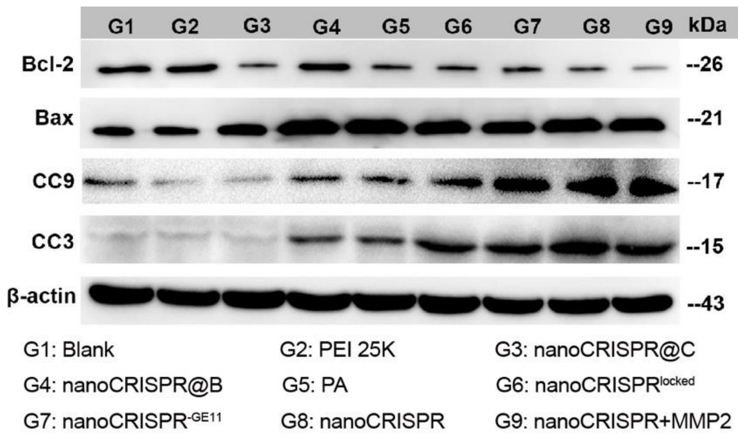

Figure S13 Representative images of the Western blots assay.

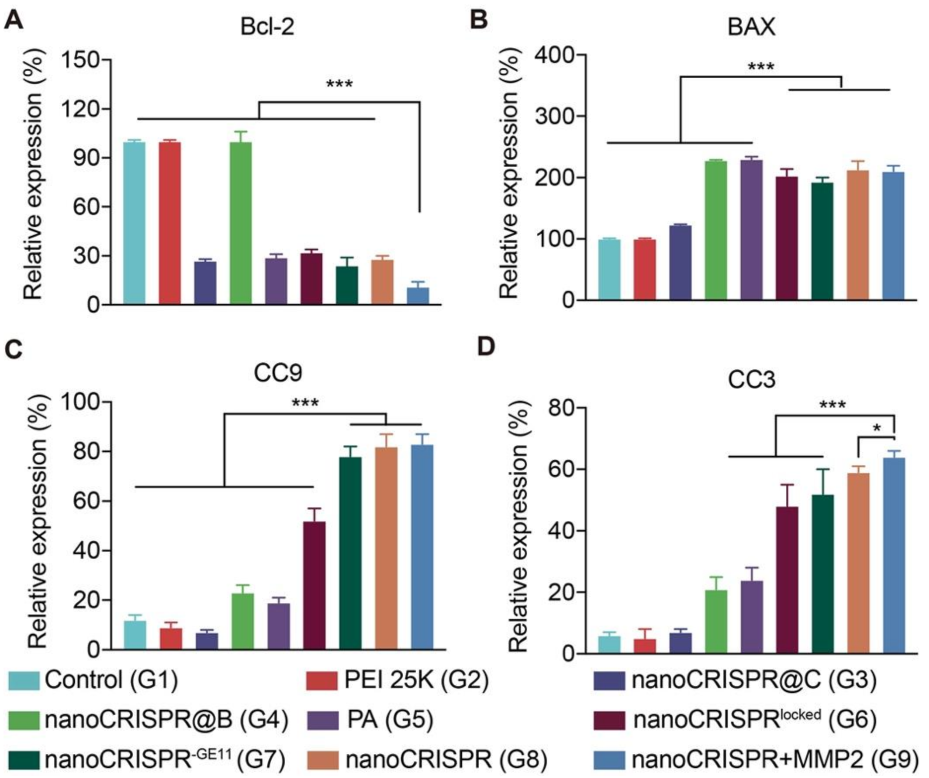

Figure S14 The statistic analysis of protein expression by one-way ANOVA tests. Data were presented as the mean ± SD (*n*=6). $^*P < 0.05$, $^{**}P < 0.01$, $^{***}P < 0.001$.

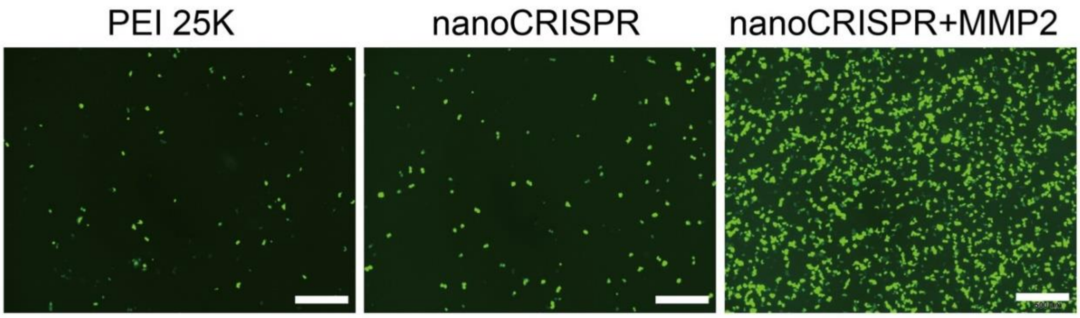

Figure S15 Fluorescence images of continuous transfection.

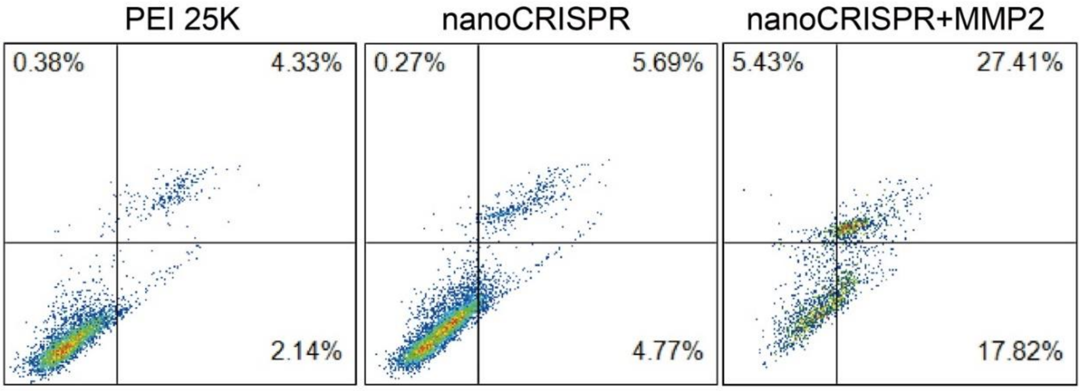

Figure S16 Flow cytometric analysis of continuous transfection.

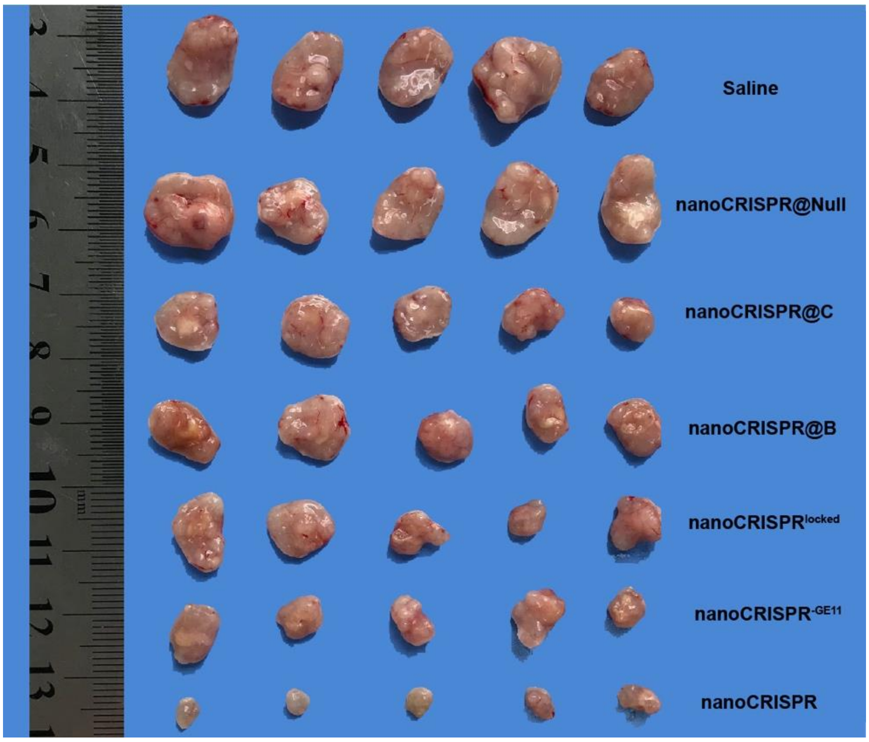

Figure S17 Representative images of tumors in different groups.

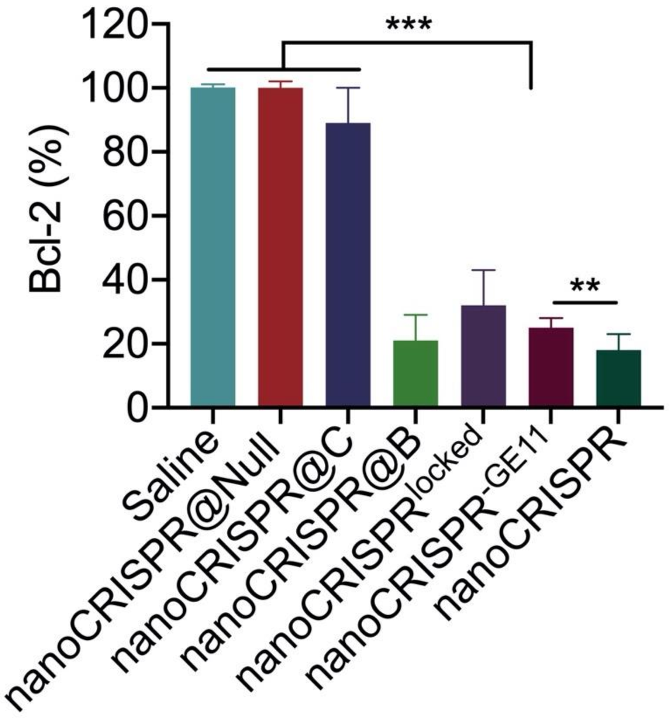

Figure S18 The statistically analysis of Bcl-2 expression using one-way ANOVA tests. Data were presented as the mean ± SD (*n*=6). *P < 0.05, **P < 0.01, ***P < 0.001.

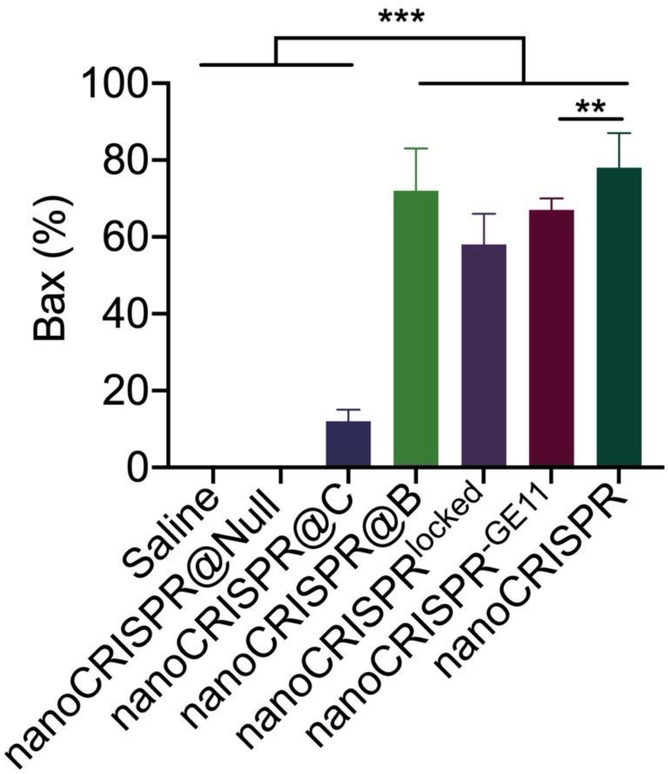

Figure S19 The statistically analysis of Bax expression using one-way ANOVA tests. Data were presented as the mean ± SD (n=6). *P < 0.05, **P < 0.01, ***P < 0.001.

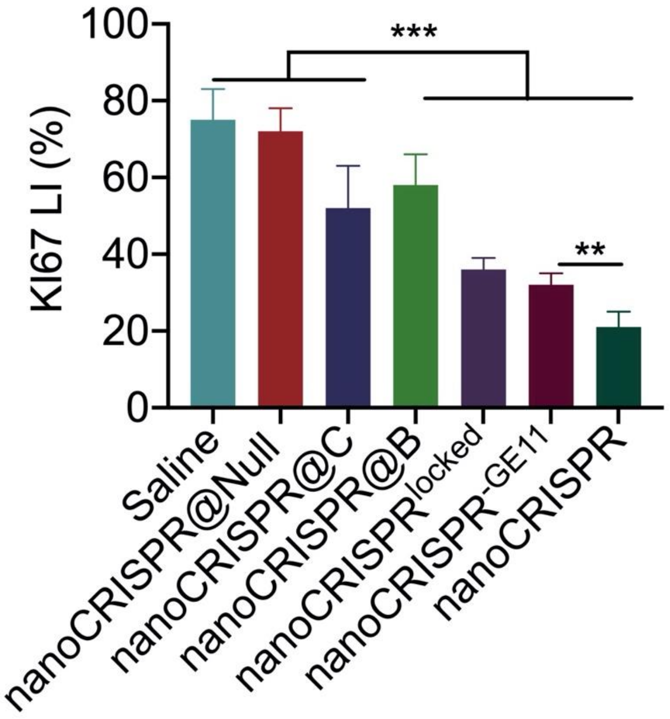

Figure S20 The statistically analysis of KI67 expression using one-way ANOVA tests. Data were presented as the mean ± SD (n=6). *$P < 0.05$, **$P < 0.01$, ***$P < 0.001$.

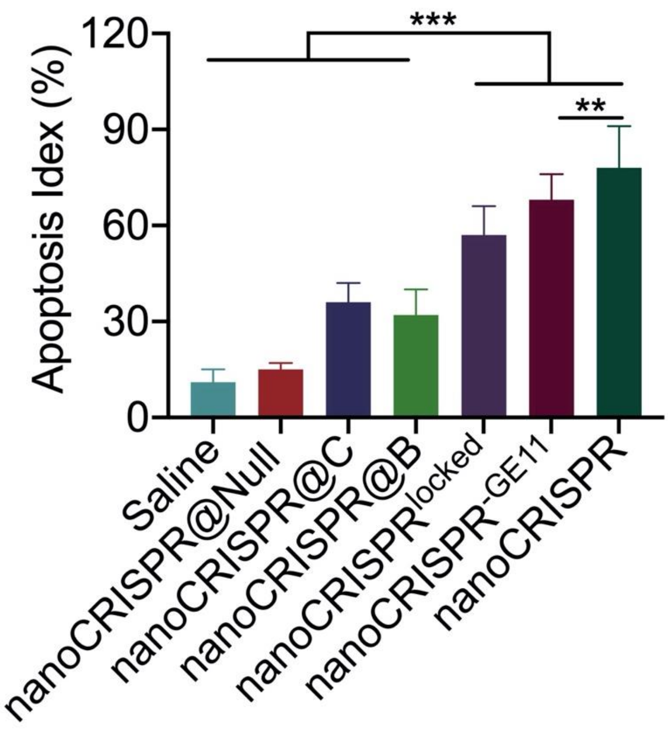

Figure S21 The statistically analysis of tumor apoptosis using one-way ANOVA tests. Data were presented as the mean ± SD (*n*=6). *$P < 0.05$, **$P < 0.01$, ***$P < 0.001$.

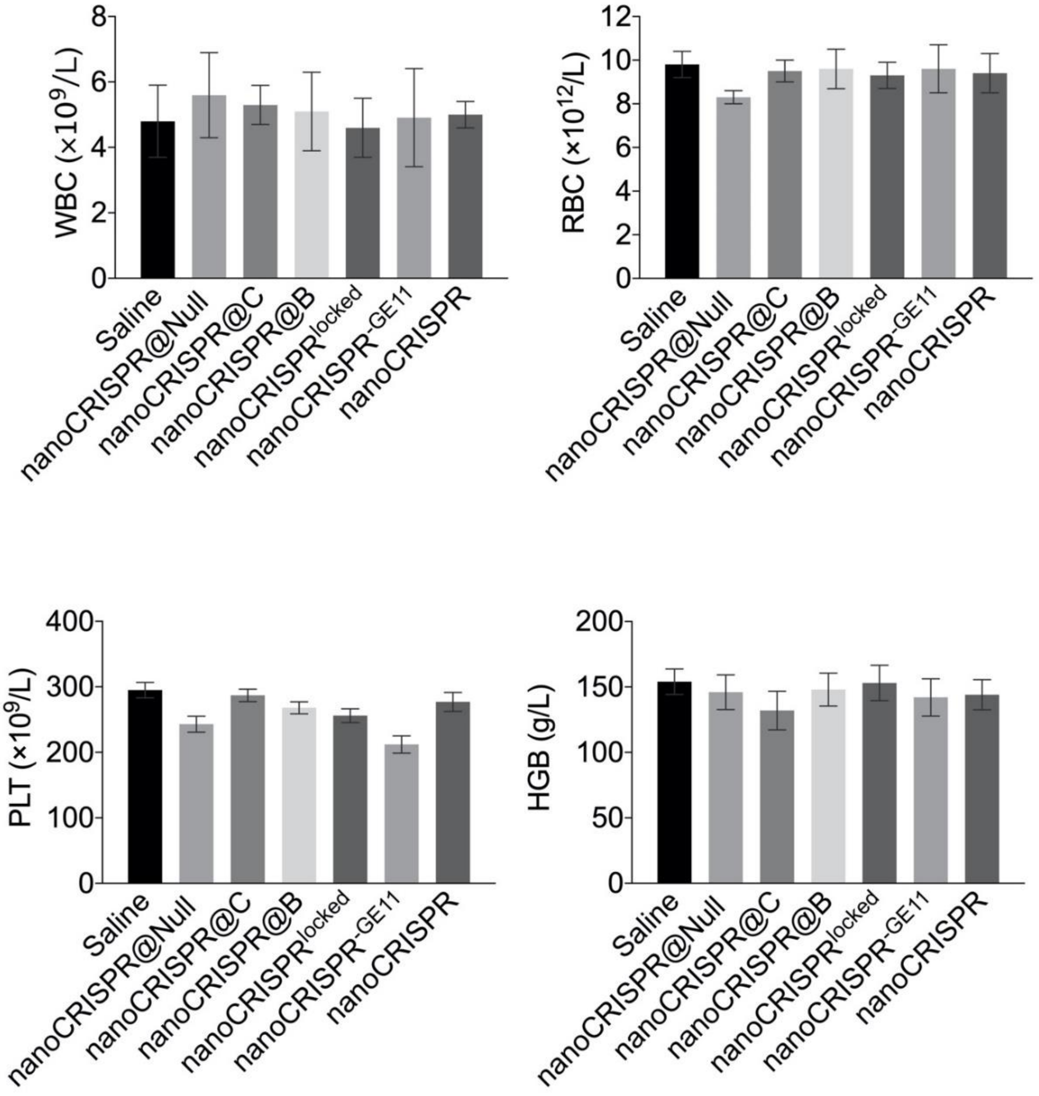

Figure S22 Blood chemistry analysis of mice after different treatments.

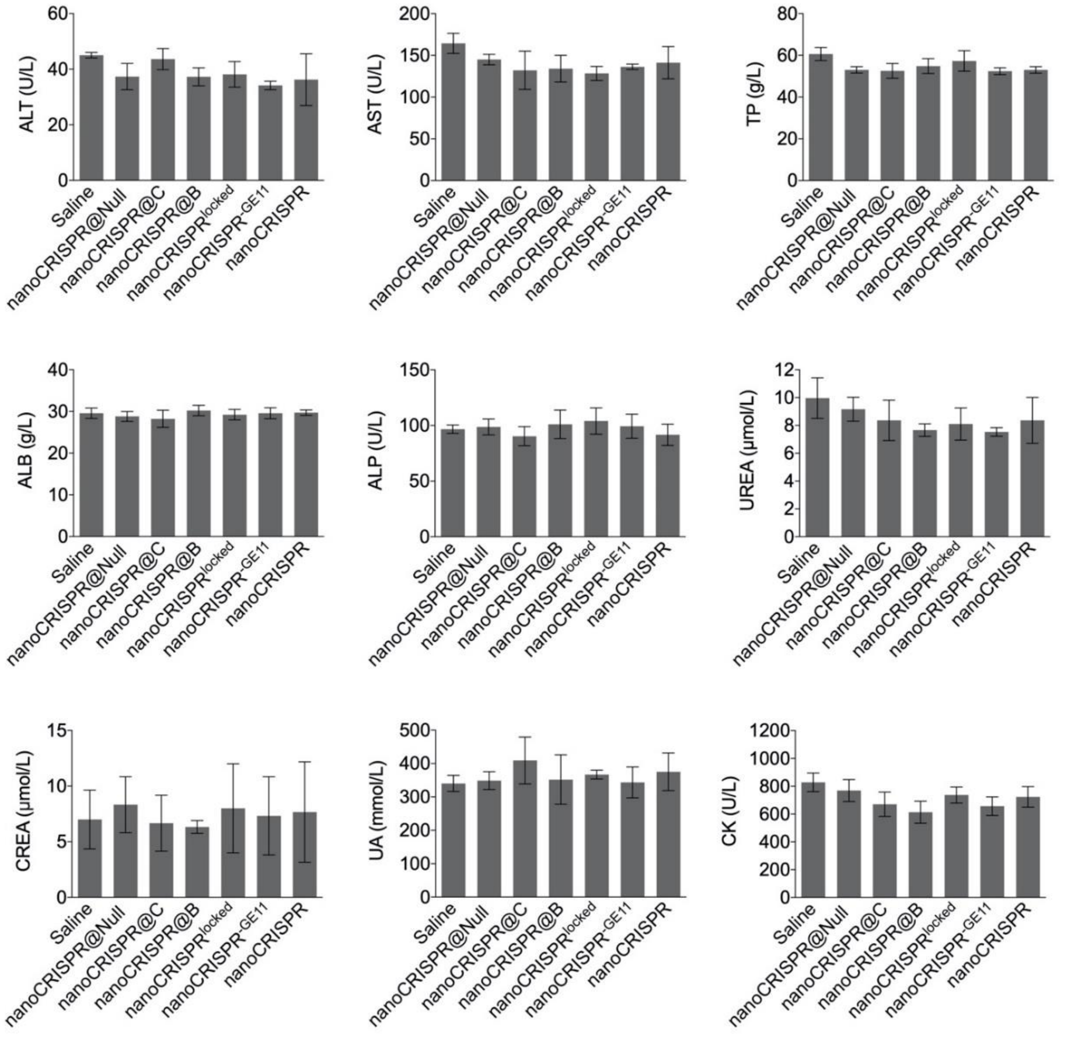

Figure S23 Complete blood count of mice after different treatments.
